# National surveillance of stroke quality of care and outcomes by applying post-stratification survey weights on the Get With The Guidelines-Stroke patient registry

**DOI:** 10.1186/s12874-021-01214-z

**Published:** 2021-02-04

**Authors:** Boback Ziaeian, Haolin Xu, Roland A. Matsouaka, Ying Xian, Yosef Khan, Lee S. Schwamm, Eric E. Smith, Gregg C. Fonarow

**Affiliations:** 1 Division of Cardiology, David Geffen School of Medicine at University of California, 10833 LeConte Avenue, Room A2-237 CHS, Los Angeles, CA 90095-1679 USA; 2 Division of Cardiology, Veteran Affairs Greater Los Angeles Healthcare System, Los Angeles, California USA; 3 Duke Clinical Research Institute, Durham, North Carolina UK; 4 Department of Biostatistics and Bioinformatics, Duke University, Durham, North Carolina UK; 5 Department of Neurology, Duke University Medical Center, Durham, North Carolina UK; 6 Healthcare Quality Research and Bioinformatics, American Heart Association, Dallas, TX USA; 7 Department of Neurology, Comprehensive Stroke Center Massachusetts General Hospital and Harvard Medical School, Boston, MA USA; 8 Department of Clinical Neurosciences and Hotchkiss Brain Institute, University of Calgary, Calgary, Alberta Canada; 9 Ahmanson-UCLA Cardiomyopathy Center, University of California, Los Angeles Medical Center, Los Angeles, California USA

**Keywords:** Epidemiology, Ischemic stroke, Quality and outcomes, Health services, Bayesian analysis, Population surveillance

## Abstract

**Background:**

The U.S. lacks a stroke surveillance system. This study develops a method to transform an existing registry into a nationally representative database to evaluate acute ischemic stroke care quality.

**Methods:**

Two statistical approaches are used to develop post-stratification weights for the Get With The Guidelines-Stroke registry by anchoring population estimates to the National Inpatient Sample. Post-stratification survey weights are estimated using a raking procedure and Bayesian interpolation methods. Weighting methods are adjusted to limit the dispersion of weights and make reasonable epidemiologic estimates of patient characteristics, quality of hospital care, and clinical outcomes. Standardized differences in national estimates are reported between the two post-stratification methods for anchored and non-anchored patient characteristics to evaluate estimation quality. Primary measures evaluated are patient and hospital characteristics, stroke severity, vital and laboratory measures, disposition, and clinical outcomes at discharge.

**Results:**

A total of 1,388,296 acute ischemic strokes occurred between 2012 and 2014. Raking and Bayesian estimates of clinical data not available in administrative data are estimated within 5 to 10% of margin for expected values. Median weight for the raking method is 1.386 and the weights at the 99th percentile is 6.881 with a maximum weight of 30.775. Median Bayesian weight is 1.329 and the 99th percentile weights is 11.201 with a maximum weight of 515.689.

**Conclusions:**

Leveraging existing databases with patient registries to develop post-stratification weights is a reliable approach to estimate acute ischemic stroke epidemiology and monitoring for stroke quality of care nationally. These methods may be applied to other diseases or settings to better monitor population health.

**Supplementary Information:**

The online version contains supplementary material available at 10.1186/s12874-021-01214-z.

## Background

The Institute of Medicine’s (IOM) report entitled *A Nationwide Framework for Surveillance of Cardiovascular and Chronic Lung Diseases* highlights the lack of systems to monitor the incidence and prevalence of preventable diseases at the national level [1]. While the U.S. mandates standardized reporting of causes of death through the National Vital Statistics system, comparable systems are not available for incident disease and the assessment of healthcare quality [2]. The IOM’s report recommends that surveillance systems be created to track progress on cardiovascular burden and inform efforts to reduce disease burden. Since the IOM’s publication in 2011, robust disease surveillance systems for cardiovascular disease have not been developed in the U.S. The glaring need to build such a surveillance system continues to be emphasized [2]. Systematically integrating various paper and electronic health record systems across the U.S. remains an insurmountable task. For this study, we sought to overcome these challenges by integrating two existing data sources for future epidemiologic and outcomes research work related to acute ischemic stroke.

A non-representative database may be transformed into a representative one if appropriate post-stratification weights are estimated to rebalance over and under-represented segments of a target population of interest [3]. Statistical methods may be used to post-stratify non-random sample observations and approximate true target population estimates.

In the U.S., the best estimates for the incidence and utilization of hospital services are publicly available through databases sponsored by the Agency for Healthcare Research and Quality’s Healthcare Cost and Utilization Project [4]. The National Inpatient Sample (NIS) is a structured random sample of U.S. hospitalizations that is then weighted to represent national hospital utilization. However, the database does not include detailed clinical data such as stroke severity, laboratory data, medical treatments received, and patient reported outcomes. A few community cohort and case-control studies are currently featured in the annual American Heart Association (AHA) statistical update on heart disease and stroke statistics, but are not nationally representative and inadequate to measure stroke burden and quality of care nationally [5–7].

The AHA-sponsored Get With The Guidelines Program (GWTG) program includes rich clinical data for quality improvement and research analyses [8]. Yet, registries with volunteer hospitals are not proportionally representative of the entire nation [9, 10]. For this study, we implement and validate advanced post-stratification weighting methods and describe the clinical characteristics of the national acute ischemic stroke population using the AHA’s GWTG-Stroke registry. Implementation of these methods form a platform for future national surveillance and health care quality research.

## Methods

### Data source

We used the GWTG-Stroke registry from 2012 to 2014 to evaluate post-stratification weighting procedures to represent the entire U.S. acute ischemic stroke (AIS) population. In GWTG-Stroke, trained personnel abstract reliable deidentified demographic, clinical, and event information from participating hospitals using an internet-based patient management tool [8]. Identification of AIS is accurately identified and clinical variables such as admission and discharge stroke severity are systematically included, alongside detailed clinical data not available in administrative claims data alone. GWTG-Stroke includes 1300–1500 hospitals per year and details are previously described [11, 12]. Hospitals participating in the GWTG program do so on a voluntary basis. Although the GWTG program contains many small, rural and non-academic hospitals, these hospital types are under-represented compared to the overall U.S. hospitalized population [9]. Therefore, the sampling strategy does not directly estimate national AIS clinical characteristics as currently structured.

To determine the total number of AIS hospitalizations in the U.S. and marginal population characteristics for post-stratification weights, target population counts are obtained from the NIS sponsored by the Agency for Healthcare Research and Quality. For 2012 to 2014, the NIS sampled 20% of the administrative discharge records from all participating hospitals (approximately 4300 hospitals) covering 95% of the U.S. population and 94% of all community hospital discharges [13]. While the NIS may be used to understand populations rates of AIS, basic demographics, procedures, and costs, which lacks detailed clinical and outcomes data.

### Study population

The target population for the post-stratification weighting procedure is the total AIS presenting to U.S. hospitals by year. The NIS defines the AIS burden nationally stratified between the years of 2012 and 2014 and the 9 U.S. Census regions – preserving the smallest sampling unit recommended by the NIS sponsors.

### Data definitions

AIS is defined using the primary discharge diagnosis from the first listed International Classification of Diseases, Ninth Revision (ICD-9) code for each NIS hospitalization [14]. AIS is defined in GWTG-Stroke based on abstracted discharge diagnoses (online supplement, eTable 1). GWTG-Stroke uses electronic case report form-based data extraction from clinical chart review to document patient-specific comorbid conditions. The NIS diagnostic and procedure estimates are based on administrative coding of ICD-9 diagnostic and procedure codes.

### Statistical analysis

Two parallel methods are used to estimate post-stratification survey weights. Raking is an iterative procedure for minimizing the dispersion of weights for each observation relative to the average sample weight to approximate marginal counts for characteristics of interest. More recent research has advanced Bayesian interpolation statistical methods to estimate post-stratification weights and fit flexible analytic models. Both raking and the Bayesian interpolation method rely on anchoring estimates to a select characteristics shared between disparate datasets in order to correct skewed distributions. For this study, select hospital and patient characteristics are added iteratively as anchoring variables to improve skewed representation within GWTG-Stroke. The two post-stratification epidemiologic estimates regarding AIS care are contrasted.

Standardized differences for all weighted characteristics are estimated for patient and hospital characteristics (anchored and non-anchored variables). We analyze the distribution of raking and Bayesian weights with histograms and treemaps to provide a perspective on the skewed representation of the GWTG-Stroke raw sample. Iterative model development is used to select the minimal set of hospital or patient characteristics necessary to limit extreme post-stratification weights while maintaining reliable population estimates for known NIS estimates.

#### Overview of the estimation problem

Suppose we want to estimate the proportion of eligible patients for different age categories in the population. For each census division (i.e., sample *s*) and for the elements *k* in the census division, i.e., *k* ∈ *s*, we observe in the registry a number *x*_*k*_ hospitalizations, with some of them possibly under- (or over-) represented relative to the target population. Using data from the available registry, our goal is to estimate the probability sampling weight *w*_*k*_ such that

$$ \sum \limits_{k\in s}\ {\mathrm{w}}_k{x}_k={t}_x $$

where *t*_*x*_ is the observed mean for the target population from the NIS [15]. For this study, we derive the post-stratification weights *w*_*k*_ using two parallel approaches: raking and the Bayesian interpolation.

#### Raking procedure

Raking procedures are used to generate weights when known marginal counts are available for two or more categorical variable dimensions [16–18]. The raking algorithm creates an initial weight for all observations and then iteratively adjusts them to minimize the spread of weights, so no single observation is over- or under-represented in the data [17]. Therefore, if the target male population is 400,000 and the sample population is 200,000 males, an initial raking weight of 2 would apply to all observations across male sex. Raking attempts to minimize the difference between new weights and the initial weight to approximate the targeted population totals across multiple anchoring dimensions.

The initial or base weight *d*_*k*_ based on the population size, such that *d*_*k*_ multiplied by the sample size equals the population size. The goal of a raking procedure is to minimize the sum of the difference between the new weights (*w*_*k*_) and the base weight (*d*_*k*_) [15]. Raking attempts to estimate a determined *t*_*x*_ target while minimizing the average weight distance from the base weight.

$$ Average\ weight\ distance=\sum \limits_{k\in s}{\left({w}_k-{d}_k\right)}^2/{d}_k $$

Typically, weighted variance estimation (i.e. the Horvitz-Thomson estimator) of structured data accounts for the inclusion probability of sampled data from a population [16]. Post-stratification variance estimation with raking uses an additive analysis of variance (ANOVA) of the residuals to fit the model [17, 19]. Variables available in both GWTG-Stroke and the NIS are selected as anchoring variables to generate the raking weights using SAS 9.4 (SAS Institute, Inc., Cary, North Carolina). Shortcomings of this frequentist approach to probability weight generation remain. Statistical assumptions may not hold for variance estimation, especially for testing interactions and small-area estimation [20]. This procedure may also create negative weights in certain constrained data situations [21]. Variables evaluated for raking included: age quartiles, sex, race/ethnicity, region, payer, hospital bed size, hospital ownership (government, private non-profit, private investor-owned) and rural/urban status.

#### Bayesian population interpolation

The Bayesian population interpolation approach frames post-stratification weights as estimated from the posterior distribution of anchoring variables for the target population (i.e. total U.S. AIS population). The Bayesian model allows for greater flexibility and the ability to integrate information from multiple sources that account for the known marginal and joint distributions of various population characteristics over time. For this study, only the NIS is required to calibrate post-stratification weights. The observed proportions from GWTG-Stroke are Bayesian prior information within the model and are non-representative of the target population.

The Bayesian model estimates post-stratification weights when integrating prior and posterior information for the anchored variables. The observed GWTG-Stroke dataset (Bayesian prior) when fit to the marginal distribution of the anchoring characteristics generates post-stratification weights [22, 23]. The fundamental model is described as such: let *p*_*m*_ represent the observed proportion for a given variable *m* for subgroup with *φ*_*m*_ being the true population proportion. Observed counts are represented by the sample size multiplied by the observed proportion (n_s_p_m_)). Next, we build a multinomial observational model for adjusting the observed and known subgroup proportions:

1 $$ {n}_s{p}_m\sim multinomial\left({\varphi}_m{n}_s^r\right) $$

where *n*_*s*_ represents the size of the sample and *n*_*s*_*p*_*m*_ is the number of patients that fall within different sub-categories (i.e. *m = 1, 2, 3)* of the sample of patients (for which the observed numbers are the naïve estimates). The number ${n}_s^r$ is the precision of the sampling distribution, which we specify in the application based on *n*_*s*_. Under this model, the expected value of the proportion *p*_*m*_ is thus *φ*_*m*_. Finally, for a given cell, *φ*_*m*_ = *A*_*m*_*π* , where *π* is the true (unknown) cell population and *A*_*m*_ is an indicator matrix whose component are equal to 1 when the observed cell is not empty and 0 otherwise.

For each year, the anchoring covariates form joint distributions between the observed GWTG-Stroke observations and target population proportions. The conjugate of the multinomial distribution *π*_*τ*_~*Dir*(*π*_*τ* − 1_, *n*^*h*^) are Dirichlet models linked through a stochastic relationship (represented by the indexes *τ*) between each GWTG-Stroke observation and the marginal and joint distributions for the target AIS population derived from the NIS [24]. The hyperparameter *n*^*h*^ models the degree of pooling across available registries to which we assign a low prior. The Bayesian model includes permutations of all anchored variable combinations as population subgroups. For variable combinations where GWTG-Stroke lacked observations, non-zero cell populations (i.e., related *n*^*h*^) are used for estimation. We assume a flat prior for the GWTG-Stroke observations to approximate the target population characteristics from the NIS. Once the posteriors of *φ*_*m*_ = *A*_*m*_*π* are calculated, we determine the weights *w*_*k*_ as *w*_*k*_ = *p*_*m*_, using the equality [1]. All Bayesian analyses are performed in R 3.6.1 (R Foundation, Vienna Austria). Permission for this analysis was granted through the Duke Clinical Research Institute IRB.

## Results

**Fig. 1:**
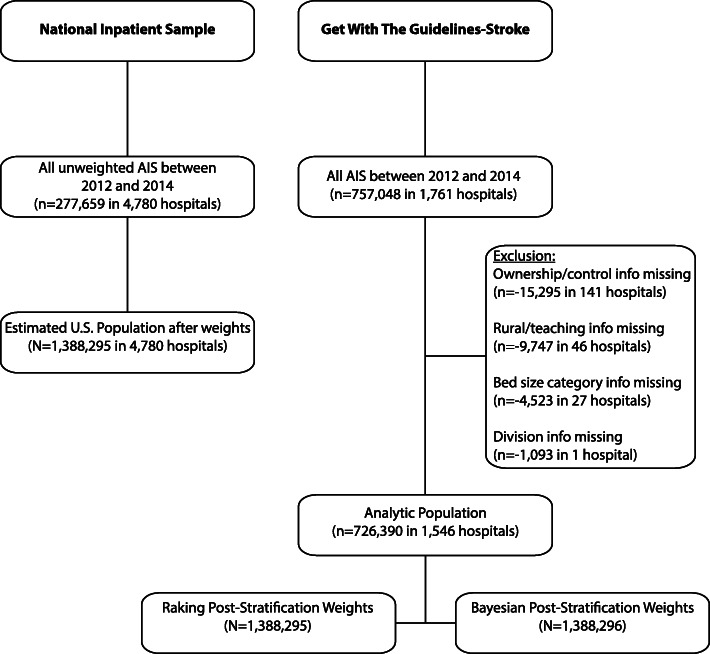
Flow Chart of study population inclusion from the National Inpatient Sample and the Get With The Guidelines-Stroke registry program. AIS = Acute Ischemic Stroke

A total 1761 hospitals are included in the GWTG-Stroke registry between 2012 and 2014. We excluded hospitals in which hospital characteristics of interest are not fully recorded in the database. The final cohort included 726,390 patients across 1546 hospitals representing the raw GWTG-Stroke cohort prior to weighting (Fig. 1 and Online Supplement eTable 2, 3, 4).

Initially, we attempted a parsimonious model to generate the weights using only select hospital characteristics: ownership, rural/teaching, and bed size stratified by Census division. After observing inadequate representation for select race/ethnic minorities, a decision was made to include patient-level race/ethnicity to derive post-stratification weights. Weights are unique for each hospitalization observed in GWTG-Stroke. The final raking and Bayesian post-stratification weight models used hospital characteristics for ownership, rural/urban and teaching status, bed size followed by race/ethnicity at the patient-level.

There were an estimated 1,388,296 AIS hospitalizations between 2012 to 2014 in the U.S. For the raking method, anchored characteristics in the weighted GWTG-Stroke sample matched the exact population totals estimated from the NIS. This is to be expected unless matching two or more marginal characteristics is mathematically prohibitive (Table 1). The Bayesian method generates population totals with no more than 5–10% variance of the NIS estimates. While the NIS estimates AIS presented to rural hospitals 10.29% of the time, the GWTG-Stroke unweighted representation is 3.49% and after post-stratification using Bayesian derived weights is 6.02%, which is 44% lower than expected. Age distributions for both methods are extremely similar. Sex, race/ethnicity, health insurance status, and comorbidities, vital and laboratory measurements, arrival information and hospital characteristics are also similar between the raking and Bayesian methods. Post-stratification estimates stratified by year and U.S. Division are available in the Online Supplement eTable 5 through 7.

**Table 1:** Patient characteristics in the Get With The Guideline – Stroke after post-stratification weights using raking or Bayesian approach

	GWTG Unweighted	NIS	GWTG Raking Weights	GWTG Bayesian Weights	Standardized Differences, %		
	*N* = 726,390	*N* = 1,388,295	N = 1,388,295	N = 1,388,296	NIS vs Raking	NIS vs Bayesian	Raking vs Bayesian
**Hospital Characteristics**							
*Census divisions					0.0	3.9	3.9
Division 1 New England	40,284 (5.55)	59,960 (4.32)	59,960 (4.32)	61,260 (4.41)			
Division 2 Mid-Atlantic	141,026 (19.41)	190,045 (13.69)	190,045 (13.69)	191,365 (13.78)			
Division 3 East North Central	98,744 (13.59)	215,585 (15.53)	215,585 (15.53)	217,076 (15.64)			
Division 4 West North Central	41,280 (5.68)	90,955 (6.55)	90,955 (6.55)	83,707 (6.03)			
Division 5 South Atlantic	159,799 (22.00)	303,745 (21.88)	303,745 (21.88)	314,341 (22.64)			
Division 6 East South Central	39,350 (5.42)	114,565 (8.25)	114,565 (8.25)	107,499 (7.74)			
Division 7 West South Central	66,934 (9.21)	158,475 (11.42)	158,475 (11.42)	160,105 (11.53)			
Division 8 Mountain	37,864 (5.21)	72,795 (5.24)	72,795 (5.24)	66,735 (4.81)			
Division 9 Pacific	101,109 (13.92)	182,170 (13.12)	182,170 (13.12)	186,208 (13.41)			
*Hospital ownership					0.0	5.6	5.6
Government	73,541 (10.12)	165,400 (11.91)	165,400 (11.91)	142,585 (10.27)			
Private, Non-Profit	579,983 (79.84)	1,034,510 (74.52)	1,034,510 (74.52)	1,063,608 (76.61)			
Private, Investor-Owned	72,866 (10.03)	188,385 (13.57)	188,385 (13.57)	182,102 (13.12)			
*Rural/teaching status					0.0	16.0	16.0
Rural	25,374 (3.49)	142,920 (10.29)	142,920 (10.29)	83,637 (6.02)			
Urban nonteaching	149,164 (20.53)	476,970 (34.36)	476,970 (34.36)	477,741 (34.41)			
Urban teaching	551,852 (75.97)	768,405 (55.35)	768,405 (55.35)	826,917 (59.56)			
*Bed Size Categories					0.0	7.4	7.4
Small	92,088 (12.68)	184,630 (13.30)	184,630 (13.30)	159,846 (11.51)			
Medium	198,454 (27.32)	379,405 (27.33)	379,405 (27.33)	357,012 (25.72)			
Large	435,848 (60.00)	824,260 (59.37)	824,260 (59.37)	871,437 (62.77)			
Primary Stroke Center	509,534 (70.15)	N/A	941,419 (67.81)	953,966 (68.71)	–	–	1.9
Comprehensive Stroke Center	110,333 (15.19)	N/A	149,156 (10.74)	179,012 (12.89)	–	–	6.7
Number of Beds, Median (IQR)	374 (243–581)	N/A	302 (195–464)	350 (205–532)	–	–	9.8
Annual Volume of IS Admissions, Median (IQR)	243 (166–382)	N/A	208 (143–318)	228 (143–361)	–	–	8.2
**Patient Characteristics**							
Age					2.4	1.5	0.8
Mean (SD)	70.49 (14.57)	70.61 (14.10)	70.47 (20.02)	70.29 (20.11)			
Age category							0.8
≤ 60	184,201 (25.36)	339,800 (24.48)	350,934 (25.28)	356,665 (25.69)			
61–70	160,447 (22.09)	302,770 (21.81)	309,032 (22.26)	309,064 (22.26)			
71–80	169,763 (23.37)	328,650 (23.67)	327,235 (23.57)	326,584 (23.52)			
> 80	211,979 (29.18)	417,075 (30.04)	401,094 (28.89)	395,981 (28.52)			
Female	368,770 (50.77)	714,159 (51.44)	704,825 (50.77)	701,281 (50.51)	1.3	1.9	0.5
*Race/Ethnicity					0.0	4.6	4.6
White	506,456 (69.72)	925,390 (66.66)	925,390 (66.66)	923,221 (66.50)			
Black	124,170 (17.09)	217,450 (15.66)	217,450 (15.66)	214,227 (15.43)			
Hispanic	46,836 (6.45)	98,615 (7.10)	98,615 (7.10)	99,818 (7.19)			
Asian & Pacific Islander	22,425 (3.09)	34,935 (2.52)	34,935 (2.52)	45,134 (3.25)			
Other	26,503 (3.65)	111,905 (8.06)	111,905 (8.06)	105,896 (7.63)			
Insurance					13.1	14.3	1.7
Private/VA/Champus/Other Insurance	140,727 (23.12)	256,085 (19.01)	259,132 (22.47)	268,964 (23.02)			
Medicaid	39,428 (6.48)	104,045 (7.72)	71,336 (6.19)	73,610 (6.30)			
Medicare	388,813 (63.88)	917,520 (68.10)	741,833 (64.32)	741,999 (63.51)			
Self Pay/No Insurance	39,722 (6.53)	69,685 (5.17)	81,042 (7.03)	83,748 (7.17)			
**Stroke Admission Year**							
2012	220,387 (30.34)	452,240 (32.58)	452,240 (32.58)	452,240 (32.58)			
2013	242,633 (33.40)	460,400 (33.16)	460,400 (33.16)	460,400 (33.16)			
2014	263,370 (36.26)	475,655 (34.26)	475,655 (34.26)	475,655 (34.26)			
**Medical History**							
Atrial Fibrillation/Flutter	172,120 (23.76)	343,981 (24.78)	318,990 (23.05)	320,231 (23.14)	4.0	3.8	0.2
Previous Stroke/TIA	222,336 (30.99)	N/A	429,240 (31.31)	423,422 (30.94)	–	–	0.8
CAD/Prior Myocardial Infarction	176,850 (24.65)	378,739 (27.28)	341,816 (24.93)	339,277 (24.79)	5.4	5.7	0.3
Diabetes Mellitus	243,745 (33.97)	553,176 (39.85)	473,934 (34.57)	469,364 (34.29)	10.9	11.5	0.6
Peripheral Vascular Disease	33,481 (4.67)	142,639 (10.27)	64,133 (4.68)	64,556 (4.72)	21.4	21.2	0.2
Hypertension	548,231 (76.41)	1,149,625 (82.81)	1,049,345 (76.54)	1,043,025 (76.21)	15.6	16.4	0.8
Smoker	133,412 (18.59)	433,520 (31.23)	258,994 (18.89)	259,894 (18.99)	28.8	28.5	0.3
Dyslipidemia	325,549 (45.37)	797,295 (57.43)	615,319 (44.88)	613,296 (44.81)	25.3	25.4	0.1
Heart Failure	66,449 (9.26)	199,810 (14.39)	125,027 (9.12)	126,046 (9.21)	16.4	16.1	0.3
Prosthetic Heart Valve	9147 (1.27)	20,590 (1.48)	16,757 (1.22)	17,899 (1.31)	2.3	1.5	0.8
Obesity/Overweight	84,405 (11.76)	151,915 (10.94)	148,136 (10.80)	159,219 (11.63)	0.4	2.2	2.6
Chronic Renal Insufficiency	40,204 (5.60)	200,960 (14.48)	74,183 (5.41)	74,472 (5.44)	30.6	30.5	0.1
**Vital and Laboratory Measurements**							
SBP mmHg, Mean (SD)	157.02 (30.09)	N/A	157.51 (41.69)	157.35 (41.69)	–	–	0.4
BMI, Median (IQR)	27.2 (23.8–31.6)	N/A	27.3 (23.8–31.7)	27.3 (23.8–31.6)	–	–	0.0
HbA1c, % Mean (SD)	6.71 (1.89)	N/A	6.77 (2.57)	6.74 (2.6)	–	–	1.4
Blood Glucose mg/dL, Mean (SD)	142.48 (70.78)	N/A	143.65 (99.06)	143.42 (99.34)	–	–	0.3
Serum Creatinine mg/dL, Median (IQR)	1 (0.8–1.3)	N/A	1 (0.8–1.3)	1 (0.8–1.3)	–	–	0.2
**Arrival Information**							
Arrival Mode: EMS	328,713 (49.63)	N/A	615,016 (48.88)	608,291 (48.43)	–	–	0.9
Ambulatory Status at Admission					–	–	0.7
Unable to ambulate	140,461 (32.84)	N/A	258,705 (31.75)	261,489 (31.79)			
With assistance from person	117,069 (27.37)	N/A	228,196 (28.01)	232,655 (28.28)			
Able to ambulate independently	170,187 (39.79)	N/A	327,796 (40.24)	328,418 (39.93)			
On-time Arrival (non-holiday weekday 7 am-6 pm)	351,852 (48.44)	N/A	680,317 (49.00)	676,280 (48.71)	–	–	0.6
Initial NIHSS Score (0–42)					–	–	0.5
Median (IQR)	4 (1–9)	N/A	4 (1–9)	4 (1–9)			
Mean (SD)	6.7 (7.57)	N/A	6.63 (10.43)	6.68 (10.41)			
**Medications Prior to Admission**							
Antiplatelets	315,626 (49.64)	N/A	597,965 (49.49)	593,907 (49.15)	–	–	0.7
Anticoagulants	70,885 (15.87)	N/A	131,611 (15.49)	132,891 (15.56)	–	–	0.2
Antihypertensives	411,912 (69.26)	N/A	778,405 (69.30)	783,141 (69.14)	–	–	0.3
Cholesterol-Reducers	320,192 (44.35)	N/A	607,248 (43.98)	600,088 (43.55)	–	–	0.9
Diabetic Medications	156,575 (26.98)	N/A	302,257 (27.61)	301,123 (27.37)	–	–	0.5
**Outcomes**							
Length of Stay, (days), Median (IQR)	4 (2–6)	3 (2–6)	4 (2–6)	4 (2–6)	–	–	0.8
Stroke Unit Admission	394,102 (73.18)		710,891 (70.40)	710,406 (69.84)	–	–	1.2
Discharge Disposition							0.9
Home	343,284 (47.26)	679,755 (48.96)	663,414 (47.79)	660,288 (47.56)	2.3	2.7	
Home Hospice	10,019 (1.38)	N/A	19,336 (1.39)	19,701 (1.42)	–	–	
Hospice Facility	22,950 (3.16)	N/A	43,410 (3.13)	43,532 (3.14)	–	–	
Acute Care Facility	14,739 (2.03)	40,225 (2.90)	33,304 (2.40)	34,595 (2.49)	–	–	
Other Health Care Facility	297,278 (40.93)	592,875 (42.71)	558,726 (40.25)	558,770 (40.25)	4.9	4.9	
Left Against Medical Advice	4954 (0.68)	10,720 (0.77)	9644 (0.69)	9459 (0.68)	–	–	
Expired (in-hospital mortality)	32,540 (4.48)	62,430 (4.50)	59,108 (4.26)	60,650 (4.37)	1.2	0.6	
Discharge Disposition - Other Facilities					–	–	1.5
Skilled Nursing Facility	128,134 (43.40)	N/A	247,379 (44.57)	243,845 (43.93)			
Inpatient Rehabilitation Facility	155,283 (52.60)	N/A	284,112 (51.19)	286,456 (51.61)			
Long Term Care Hospital	6322 (2.14)	N/A	11,988 (2.16)	12,680 (2.28)			
Intermediate Care facility	2831 (0.96)	N/A	6238 (1.12)	6408 (1.15)			

*Characteristic used to anchor post-stratification weights

*GWTG* Get With The Guidelines, *UW* unweighted, *W* weighted, *TIA* transient ischemic attack, *CAD* coronary artery disease, *HbA1C* hemoglobin A_1C_, *EMS* emergency medical services

The NIS does not provide any clinical data such as medication lists, vitals and laboratory measurements, stroke severity and certain discharge disposition data. The NIS definitions for health insurance status did not align with the GWTG definitions, and therefore were not included in the Table 1. In GWTG, there are small differences in the prevalence of comorbidities between the raking and Bayesian weighting methods. NIS comorbidities are based on administrative coding only while GWTG-Stroke is based on chart abstraction. There are minimal differences in summary vital and laboratory measurement, arrival information, baseline medication usage rates, and inpatient outcomes between the two weighting approaches. On admission we note that 49.2% of stroke patients nationally are using antiplatelet medications, 15.5% anticoagulants, 69.1% anti-hypertensives, 43.6% cholesterol lowering medications, 27.4% diabetic medications. With respect to disposition, 47.6% of patients are discharged home 40.2% to transitional care facilities, and 4.6% with hospice-related services.

**Fig. 2:**
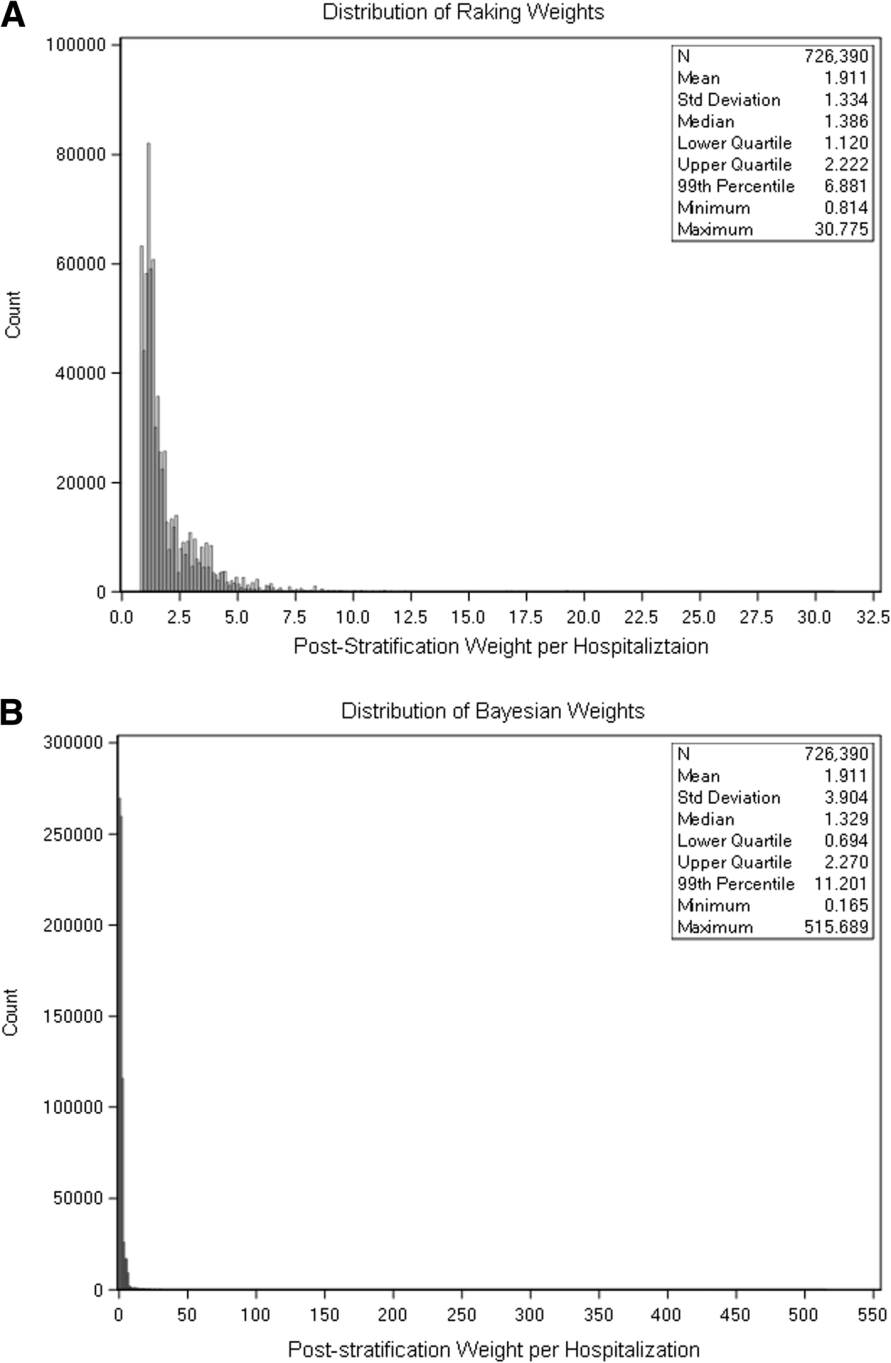
Distribution of raking and Bayesian weights. **a**: Distribution of raking derived post-stratification weights. **b**: Distribution of Bayesian post-stratification weights. Raking and Bayesian weights using hospital characteristics and patient-level race/ethnicity

For the raked post-stratification weights, the median weight is 1.386 and the weights at the 99th percentile is 6.881 with a maximum weight of 30.775 for individual GWTG-Stroke observations (Fig. 2 A and Online Supplement eFigure 1). For the Bayesian post-stratification weights, the median weight is 1.329 and the 99th percentile weights is 11.201 with a maximum weight of 515.689 (Fig. 2 B and Online Supplement eFigure 2).

**Fig. 3:**
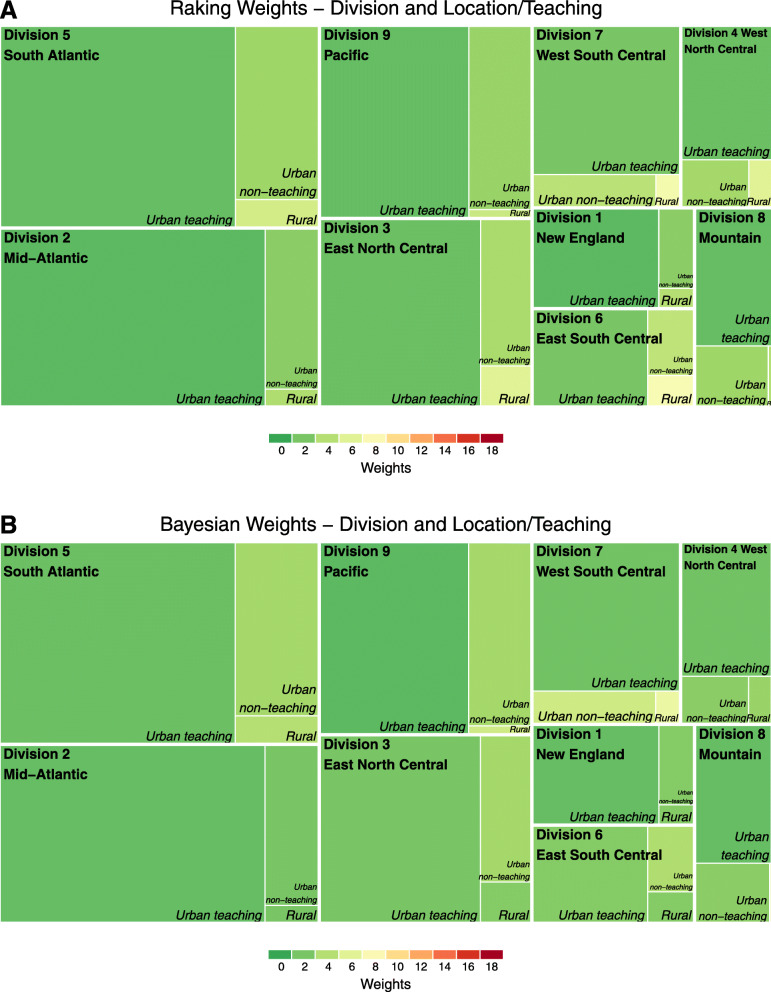
Treemaps of weighting stratified by U.S. Census division and rural/teaching hospital status. **a**, **b**: The treemaps provide a perspective of population size (box size) across region and hospital characteristic to describe the target population. The average size of the post-stratification weights used for each observation within Get With The Guideline-Stroke using the post-stratification approach. The more yellow and red regions of the treemaps highlight under-represented populations that required larger relative weights to model the target national population

**Fig. 4:**
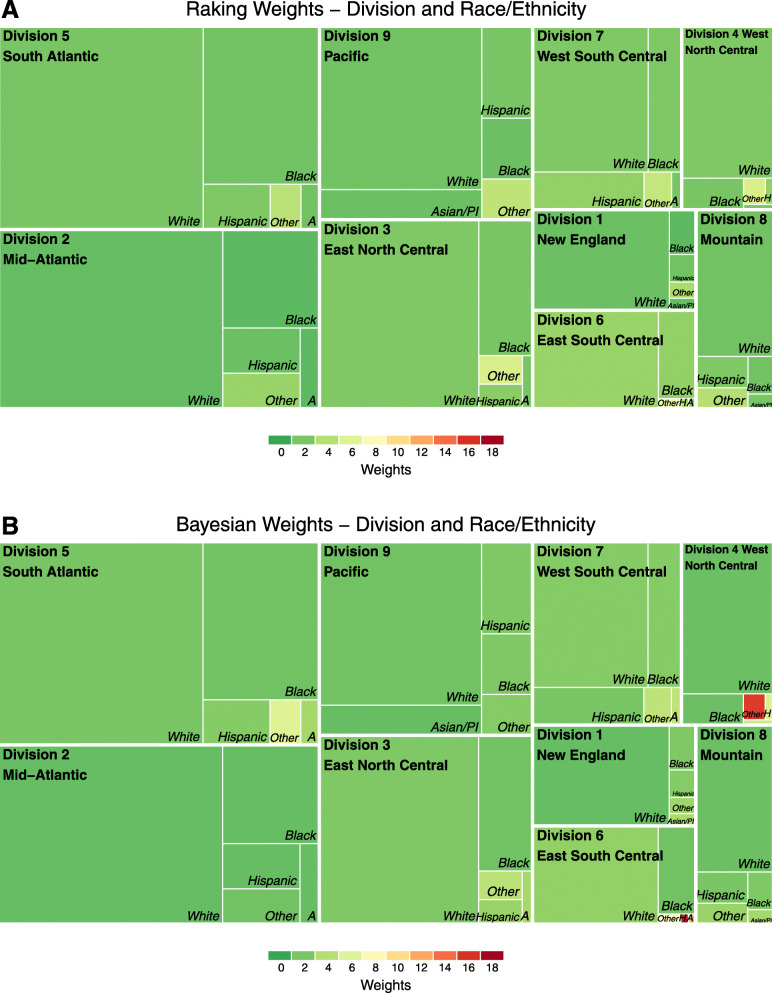
Treemaps of weighting stratified by U.S. Census division and race/ethnicity

Color treemaps permit visualization of the strata where larger weights are concentrated for select characteristics (Figs. 3 and 4). Overall, given the lower representation of rural hospitals in GWTG-Stroke, rural hospitals receive weights in the 6 to 8 range using the raking procedure. The Bayesian approach results in mostly smaller weights on average in the rural areas, however post-stratification estimates using the Bayesian method are underestimated with a standard difference of 16% compared to the raking procedure. When looking at the distribution of post-stratification weights by race/ethnicity, raking results in average weights in the 6 to 8 range for minorities in the “Other” category. Using the Bayesian method, we observe some more extreme weights for “Other” race/ethnic minorities living in the division 4 and 6.

## Discussion

The characteristics and risk factors of patients presenting with stroke nationally are not well understood given the lack of a centralized national surveillance system. Hospital care for AIS is frequently the first and last opportunity to rescue a life and reverse or prevent neurologic disability. Understanding the effectiveness of hospital systems at a national and regional level is needed to insure both consistency and timeliness in the receipt of evidence-based care. We integrate two large data systems to make better population wide clinical estimates of acute ischemic stroke in the U.S. This work demonstrates that methods exist to marry existing databases to make more reliable statistical inferences of population health and health services utilization.

The Greater Cincinnati/Northern Kentucky Stroke Study makes epidemiologic inferences using case ascertainment for an urban population to report stroke incidence rates. The population described is slightly younger, more female, has a higher representation of African-Americans, and higher rates of coronary artery disease and heart failure than is estimated from the NIS or weighted GWTG-Stroke presented (Table 2) [25–27].

**Table 2:** Comparison of patient characteristics in the National Inpatient Sample, Get With The Guidelines-Stroke, Greater Cincinnati/Northern Kentucky Stroke Study, and Reasons for Geographic and Racial Differences in Stroke Study

	NIS	GWTG-UW	GWTG-RW	GWTG-BW	GCNKSS (2010)^28^	REGARDS (2003–2007)^29^
**Patient Characteristics**						
Age, Mean (SD)	70.6 (14.1)	70.5 (14.6)	70.5 (20.0)	70.3 (20.1)	69.0 (15.3)	73 (9)
Female (%)	51.4	50.8	50.8	50.5	55.6	52.5
Race						
Black (%)	15.7	17.1	15.7	15.4	20.3	43.7
**Medical History**						
Atrial Fibrillation	24.8	18.7	23.1	23.1	22	7.7
CAD/MI (%)	27.3	24.7	24.9	24.8	31.1	40.6
Heart Failure (%)	14.4	9.3	9.1	9.2	17.2	N/A
Hypertension (%)	82.8	76.4	76.5	76.2	79.0	89.1
Diabetes Mellitus	39.9	34.0	34.6	34.3	33	37.2
Smoker (%)	31.2	18.6	18.9	19.0	28.3	21.3
Prior TIA (%)	N/A	31.0	31.3	30.9	13.4	N/A
**Vital Measurements**						
SBP mmHg, Mean (SD)	N/A	157.0 (30.1)	157.5 (41.7)	157.4 (41.7)	158.3 (31.1)	N/A
**Arrival Information**						
Baseline NIHSS, Median (IQR)	N/A	4 (1–9)	4 (1–9)	4 (1–9)	3 (1–6)	N/A

*NIS* National Inpatient Sample, *GWTG* Get With The Guidelines, *UW* unweighted, *RW* Raking weighted, *BW* Bayesian weighted, *GCNKSS* Greater Cincinnati/Northern Kentucky Stroke Study

The approach described in the present paper is a far more robust estimation of the characteristics of stroke presentation and the quality of hospital care nationally. The GWTG-Stroke patient registry captures 58% of all strokes nationally. By anchoring to the NIS, the median weights are reasonable with a median multiplier of 1.3 and very few extreme or outlier weights. The main challenges the model faced was estimation for small cohorts that are under-represented such as rural populations and other minorities in select regions of the U.S. Overall, we provide one of the best estimations for clinical characteristics expected for the entire U.S. population using GWTG-Stroke with post-stratification survey weights.

For straightforward epidemiologic estimates of clinical data from a patient registry, raking procedures are sufficient and provide good statistical stability and precision. For more complex models where additional data integration or multivariable regression modeling is required, the Bayesian approach allows greater flexibility and more direct specification of the assumptions required for measuring estimands and credible intervals.

As patient registries have expanded, advanced statistical methods are available to transform non-random samples into representative population estimates. This research demonstrated that both traditional and Bayesian methods perform well to reshape unstructured data and make inferences regarding the U.S. population. This is the first study to our knowledge that has transformed a patient registry using post-stratification weights to represent a larger population of interest. The ability to translate observations from large registries to a national scale would fill a considerable void in the surveillance of the clinical characteristics, quality of care, and outcomes for AIS hospitalizations nationally [28].

There are limitations to this work. GWTG-Stroke is a voluntary program for quality improvement. Hospitals that do not participate may be more likely to lack systems for quality improvement and therefore measures of the timeliness or completeness of AIS treatment may be biased in a favorable direction. Coding accuracy of comorbid conditions remains an issue for both administrative data from the NIS and abstracted from inpatients charts in GWTG-Stroke. Large post-stratification weights are applied to under-represented patient populations such as those in rural areas and race/ethnic minorities. Applying these methods to smaller sizes may generate less reliable estimates and may not adequately capture the diversity in patient populations. Given there is no gold standard to compare certain statistics we estimated for the U.S. AIS population, we cannot reliably test any biases that might have arisen based on the two approaches used to generate post-stratification weights. These weights are generated retrospectively, but the same methods will allow for prospective post-stratification and continuous calibration with changes in secular trends of both stroke presentation and GWTG-Stroke center participation.

## Conclusion

As healthcare in the U.S. is decentralized, there are immense practical and financial obstacles to building national or regional AIS surveillance systems. Leveraging existing patient registries such as GWTG-Stroke and applying post-stratification weights to reshape unstructured data is an efficient means of providing population surveillance of clinical measurements and outcomes not easily measured otherwise. Both raking and Bayesian approaches provide reasonably accurate estimates for describing health service utilization and the quality of care from a national perspective. We have provided a demonstration for how future researchers may approach non-survey data to achieve better representation of target population of interest. Both the raking and Bayesian interpolation methods of generating post-stratification weights may be applied to more advanced statistical modeling approaches to improve population wide inference and the surveillance of health care quality and outcomes.

## Supplementary Information

**Additional file 1: eTable 1:** ICD-9-CM diagnostic codes used to identify primary acute ischemic stroke hospitalizations in the National Inpatient Sample. **eTable 2:** Number of hospitals participating in Get With The Guidelines-Stroke per year of analysis. **eTable 3:** Population totals and proportions by U.S. Census Division for the raw Get With the Guideline-Stroke registry patients included in the final analysis. **eTable 4:** National characteristics from Table 1 with point estimates with 95% confidence intervals. **eTable 5:** Characteristics of ischemic stroke patients by year using raked post-stratification weighting by year to the U.S. Population. **eTable 6:** Characteristics of ischemic stroke patients by year using the Bayesian (flat prior) post-stratification weighting model by year to the U.S. Population. **eTable 7:** National Characteristics of ischemic stroke stratified by U.S. Division using Bayesian post-stratification weights for 2014. **eFigure 1**: Distribution of raking post-stratification weights stratified by year. **eFigure 2:** Distribution of Bayesian post-stratification weights stratified by year.

## Data Availability

Applications for access to protected health information in the registry are available to investigators through the Get With The Guideline – Stroke Registry program. https://www.heart.org/en/professional/quality-improvement/quality-research-and-publications/national-level-program-data-research-opportunities

## Abbreviations

AHA: American Heart Association
AIS: Acute Ischemic Stroke
ANOVA: Analysis of variance
GWTG: Get With The Guidelines
ICD-9: International Classification of Diseases, Ninth Revision
IOM: Institute of Medicine
NIS: National Inpatient Sample
