# Supplementary material for: National surveillance of stroke quality of care and outcomes by applying post-stratification survey weights on the Get With The Guidelines-Stroke patient registry
Source: BMC Med Res Methodol. 2021 Feb 4;21:23. doi: 10.1186/s12874-021-01214-z (PMC7863276; doi:10.1186/s12874-021-01214-z)
Supplement: Supplementary file 1 — Additional file 1: eTable 1: ICD-9-CM diagnostic codes used to identify primary acute ischemic stroke hospitalizations in the National Inpatient Sample. eTable 2: Number of hospitals participating in Get With The Guidelines-Stroke per year of analysis. eTable 3: Population totals and proportions by U.S. Census Division for the raw Get With the Guideline-Stroke registry patients included in the final analysis. eTable 4: National characteristics from Table 1 with point estimates with 95% confidence intervals. eTable 5: Characteristics of ischemic stroke patients by year using raked post-stratification weighting by year to the U.S. Population. eTable 6: Characteristics of ischemic stroke patients by year using the Bayesian (flat prior) post-stratification weighting model by year to the U.S. Population. eTable 7: National Characteristics of ischemic stroke stratified by U.S. Division using Bayesian post-stratification weights for 2014. eFigure 1: Distribution of raking post-stratification weights stratified by year. eFigure 2: Distribution of Bayesian post-stratification weights stratified by year. [file 12874_2021_1214_MOESM1_ESM.docx]

**Supplementary Online Content**

Ziaeian B, Matsouaka RA, Xu H, et. al. National Surveillance of Stroke Quality of Care and Outcomes using Post-Stratification Survey Weights on the Get With The Guidelines Stroke Patient Registry.

**eTable 1** ICD-9-CM diagnostic codes used to identify primary acute ischemic stroke hospitalizations in the National Inpatient Sample

**eTable 2** Number of hospitals participating in Get With The Guidelines-Stroke per year of analysis.

**eTable 3** Population totals by U.S. Census Division for the raw Get With the Guideline-Stroke registry patients included in the final analysis.

**eTable 4** National characteristics from Table 1 with point estimates with 95% confidence intervals.

**eTable 5**  Characteristics of ischemic stroke patients by year using raked post-stratification weighting by year to the U.S. Population.

**eTable 6** Characteristics of ischemic stroke patients by year using the Bayesian (flat prior) post-stratification weighting model by year to the U.S. Population.

**eTable 7** National Characteristics of ischemic stroke stratified by U.S. Division using Bayesian post-stratification weights for 2014.

**eFigure 1** Distribution of raking post-stratification weights stratified by year.

**eFigure 2** Distribution of Bayesian post-stratification weights stratified by year.

**eTable 1:** ICD-9-CM diagnostic codes used to identify primary acute ischemic stroke hospitalizations in the National Inpatient Sample**.**

| ICD-9-CM | Description |
| --- | --- |
| 433.01 | Occlusion and stenosis of basilar artery with cerebral infarction |
| 433.11 | Occlusion and stenosis of carotid artery with cerebral infarction |
| 433.21 | Occlusion and stenosis of vertebral artery with cerebral infarction |
| 433.81 | Occlusion and stenosis of other specified precerebral artery with cerebral infarction |
| 433.91 | Occlusion and stenosis of unspecified precerebral artery with cerebral infarction |
| 434.01 | Cerebral thrombosis with cerebral infarction |
| 434.11 | Cerebral embolism with cerebral infarction |
| 434.91 | Cerebral artery occlusion, unspecified with cerebral infarction |
| 436 | Acute, but ill-defined, cerebrovascular disease |

**eTable 2:** Number of hospitals participating in Get With The Guidelines-Stroke per year of analysis.

|  | 2012 | 2013 | 2014 |
| --- | --- | --- | --- |
| Total participating sites | **1337** | **1388** | **1450** |
| Retired sites from prior year | N/A | -64 | -45 |
| New sites from prior year | N/A | 115 | 107 |

**eTable 3:** Population totals and proportions by U.S. Census Division for the raw Get With the Guideline-Stroke registry patients included in the final analysis.

| Division | 1  New England | 2  Mid-  Atlantic | 3  East North  Central | 4  West North  Central | 5  South  Atlantic | 6  East South  Central | 7  West South  Central | 8  Mountain | 9  Pacific | Total |
| --- | --- | --- | --- | --- | --- | --- | --- | --- | --- | --- |
| Year |  |  |  |  |  |  |  |  |  |  |
| 2012 | 13,009 | 41,877 | 30,830 | 12,151 | 49,226 | 11,406 | 19,410 | 11,651 | 30,827 | 220,387 |
|  | 5.90% | 19.00% | 13.99% | 5.51% | 22.34% | 5.18% | 8.81% | 5.29% | 13.99% | 30.34% |
| 2013 | 13,260 | 47,878 | 32,493 | 13,980 | 51,881 | 13,278 | 22,403 | 12,866 | 34,594 | 242,633 |
|  | 5.47% | 19.73% | 13.39% | 5.76% | 21.38% | 5.47% | 9.23% | 5.30% | 14.26% | 33.40% |
| 2014 | 14,015 | 51,271 | 35,421 | 15,149 | 58,692 | 14,666 | 25,121 | 13,347 | 35,688 | 263,370 |
|  | 5.32% | 19.47% | 13.45% | 5.75% | 22.28% | 5.57% | 9.54% | 5.07% | 13.55% | 36.26% |
| Total | 40,284 | 141,026 | 98,744 | 41,280 | 159,799 | 39,350 | 66,934 | 37,864 | 101,109 | 726,390 |
|  | 5.55% | 19.41% | 13.59% | 5.68% | 22.00% | 5.42% | 9.21% | 5.21% | 13.92% | 100% |

**eTable 4:** National characteristics from Table 1 with point estimates with 95% confidence intervals.

|  | **GWTG**  **Unweighted** | **NIS** | **GWTG**  **Raking Weights** | **GWTG**  **Bayesian Weights** |
| --- | --- | --- | --- | --- |
|  | N=726,390 | N=1,388,295 | N=1,388,295 | N=1,388,296 |
| **Hospital Characteristics** |  |  |  |  |
| *Census divisions |  |  |  |  |
| Division 1 New England | 5.55 (5.49-5.60) | 4.32 (4.05-4.61) | 4.32 (4.29-4.35) | 4.41 (4.38-4.45) |
| Division 2 Mid-Atlantic | 19.41 (19.32-19.51) | 13.69 (13.10-14.31) | 13.69 (13.63-13.75) | 13.78 (13.73-13.84) |
| Division 3 East North Central | 13.59 (13.51-13.67) | 15.53 (14.99-16.08) | 15.53 (15.47-15.59) | 15.64 (15.58-15.70) |
| Division 4 West North Central | 5.68 (5.63-5.74) | 6.55 (6.18-6.94) | 6.55 (6.51-6.59) | 6.03 (5.99-6.07) |
| Division 5 South Atlantic | 22.00 (21.90-22.09) | 21.88 (21.28-22.49) | 21.88 (21.81-21.95) | 22.64 (22.57-22.71) |
| Division 6 East South Central | 5.42 (5.37-5.47) | 8.2.5 (7.67-8.88) | 8.25 (8.21-8.30) | 7.74 (7.70-7.79) |
| Division 7 West South Central | 9.21 (9.15-9.28) | 11.42 (10.91-11.94) | 11.42 (11.36-11.47) | 11.53 (11.48-11.59) |
| Division 8 Mountain | 5.21 (5.16-5.26) | 5.24 (4.93-5.57) | 5.24 (5.21-5.28) | 4.81 (4.77-4.84) |
| Division 9 Pacific | 13.92 (13.84-14.00) | 13.12 (12.64-13.62) | 13.12 (13.07-13.18) | 13.41 (13.36-13.47) |
| *Hospital ownership |  |  |  |  |
| Government | 10.12 (10.05-10.19) | 11.91 (11.48-12.37) | 11.91 (11.86-11.97) | 10.27 (10.22-10.32) |
| Private-Non-Profit | 79.84 (79.75-79.94) | 74.52 (73.85-75.18) | 74.52 (74.44-74.59) | 76.61 (76.54-76.68) |
| Private-Investor-Owned | 10.03 (9.96-10.10) | 13.57 (13.03-14.13) | 13.57 (13.51-13.63) | 13.12 (13.06-13.17) |
| *Rural/teaching status |  |  |  |  |
| Rural | 3.49 (3.45-3.54) | 10.29 (9.92-10.69) | 10.29 (10.24-10.35) | 6.02 (5.98-6.06) |
| Urban nonteaching | 20.53 (20.44-20.63) | 34.36 (33.64-35.08) | 34.36 (34.28-34.44) | 34.41 (34.33-34.49) |
| Urban teaching | 75.97 (75.87-76.07) | 55.35 (54.58-56.16) | 55.35 (55.27-55.43) | 59.56 (59.48-59.65) |
| *Bed Size Categories |  |  |  |  |
| Small | 12.68 (12.60-12.75) | 13.30 (12.82-13.79) | 13.30 (13.24-13.36) | 11.51 (11.46-11.57) |
| Medium | 27.32 (27.22-27.42) | 27.33 (26.65-28.02) | 27.33 (27.25-27.40) | 25.72 (25.64-25.79) |
| Large | 60.00 (59.89-60.11) | 59.37 (58.61-60.13) | 59.37 (59.29-59.45) | 62.77 (62.69-62.85) |
| Primary Stroke Center | 70.15 (70.04-70.25) | N/A | 67.81 (67.73-67.89) | 68.71 (68.64-68.79) |
| Comprehensive Stroke Center | 15.19 (15.11-15.27) | N/A | 10.74 (10.69-10.80) | 12.89 (12.84-12.95) |
| Number of Beds (mean) | 455.24 (454.48-456.00) | N/A | 383.77 (383.07-384.46) | 414.30 (413.58-415.02) |
| Annual Volume of IS Admissions (mean) | 290.56 (290.16-290.97) | N/A | 249.51 (249.14-249.89) | 269.75 (269.33-270.17) |
| **Patient Characteristics** |  |  |  |  |
| Age (mean) | 70.49 (70.45-70.52) | 70.61 (70.52-70.70) | 70.47 (70.43-70.50) | 70.29 (70.26-70.33) |
| Age category |  |  |  |  |
| ≤60 | 25.36 (25.26-25.46) | 24.48 (24.24-24.72) | 25.28 (25.21-25.35) | 25.69 (25.62-25.76) |
| 61-70 | 22.09 (21.99-22.18) | 21.81 (21.64-21.98) | 22.26 (22.19-22.33) | 22.26 (22.19-22.33) |
| 71-80 | 23.37 (23.27-23.47) | 23.67 (23.50-23.84) | 23.57 (23.50-23.64) | 23.52 (23.45-23.59) |
| >80 | 29.18 (29.08-29.29) | 30.04 (29.79-30.30) | 28.89 (28.82-28.97) | 28.52 (28.45-28.60) |
| Female | 50.77 (50.65-50.88) | 49.56 (48.36-48.76) | 50.77 (50.69-50.85) | 50.51 (50.43-50.60) |
| Race/Ethnicity |  |  |  |  |
| White | 69.72 (69.62-69.83) | 70.20 (69.55-70.85) | 66.66 (66.58-66.74) | 66.50 (66.42-66.58) |
| Black | 17.09 (17.01-17.18) | 16.50 (16.02-16.98) | 15.66 (15.60-15.72) | 15.43 (15.37-15.49) |
| Hispanic | 6.45 (6.39-6.50) | 7.48 (7.12-7.86) | 7.10 (7.06-7.15) | 7.19 (7.15-7.23) |
| Asian & Pacific Islander | 3.09 (3.05-3.13) | 2.65 (2.48-2.83) | 2.52 (2.49-2.54) | 3.25 (3.22-3.28) |
| Other | 3.65 (3.61-3.69) | 2.70 (2.49-2.93) | 8.06 (8.02-8.11) | 7.63 (7.58-7.67) |
| Insurance |  |  |  |  |
| Private/VA/Champus/Other Insurance | 23.12 (23.01-23.23) | 18.48 (18.22-18.74) | 22.47 (22.39-22.54) | 23.02 (22.95-23.10) |
| Medicaid | 6.48 (6.42-6.54) | 7.51 (7.33-7.69) | 6.19 (6.14-6.23) | 6.30 (6.26-6.34) |
| Medicare | 63.88 (63.76-64.00) | 66.21 (65.89-66.52) | 64.32 (64.23-64.41) | 63.51 (63.42-63.60) |
| Self-Pay/No Insurance | 6.53 (6.46-6.59) | 5.03 (4.88-5.18) | 7.03 (6.98-7.07) | 7.17 (7.12-7.22) |
| **Stroke Admission Year** |  |  |  |  |
| 2012 | 30.34 (30.23-30.45) | 32.58 (31.13-34.06) | 32.58 (32.50-32.65) | 32.58 (32.50-32.65) |
| 2013 | 33.40 (33.29-33.51) | 33.16 (31.68-34.67) | 33.16 (33.08-33.24) | 33.16 (33.08-33.24) |
| 2014 | 36.26 (36.15-36.37) | 34.26 (32.77-35.79) | 34.26 (34.18-34.34) | 34.26 (34.18-34.34) |
| **Medical History** |  |  |  |  |
| Atrial Fibrillation/Flutter | 23.76 (23.67-23.86) | 24.57 (24.36-24.77) | 23.05 (22.98-23.12) | 23.14 (23.07-23.21) |
| Previous Stroke/TIA | 30.99 (30.88-31.10) | N/A | 31.31 (31.23-31.39) | 30.94 (30.86-31.01) |
| CAD/Prior Myocardial Infarction | 24.65 (24.55-24.75) | 27.44 (27.23-27.65) | 24.93 (24.86-25.00) | 24.79 (24.72-24.86) |
| Carotid Stenosis | 3.63 (3.59-3.67) | 14.95 (14.77-15.14) | 3.57 (3.54-3.60) | 3.62 (3.59-3.65) |
| Diabetes Mellitus | 33.97 (33.86-34.08) | 39.22 (38.99-39-45) | 34.57 (34.49-34.65) | 34.29 (34.21-34.37) |
| Peripheral Vascular Disease | 4.67 (4.62-4.72) | 10.15 (9.98-10.31) | 4.68 (4.64-4.71) | 4.72 (4.68-4.75) |
| Hypertension | 76.41 (76.31-76.51) | 82.81 (82.61-83.01) | 76.54 (76.47-76.61) | 76.21 (76.14-76.28) |
| Smoker | 18.59 (18.50-18.68) | 31.23 (30.91-31.54) | 18.89 (18.83-18.96) | 18.99 (18.92-19.05) |
| Dyslipidemia | 45.37 (45.26-45.49) | 57.43 (57.10-57.76) | 44.88 (44.80-44.96) | 44.81 (44.73-44.89) |
| Heart Failure | 9.26 (9.19-9.33) | 14.39 (14.23-14.55) | 9.12 (9.07-9.17) | 9.21 (9.16-9.26) |
| Prosthetic Heart Valve | 1.27 (1.25-1.30) | 1.01 (0.97-1.06) | 1.22 (1.20-1.24) | 1.31 (1.29-1.33) |
| Obesity/Overweight | 11.76 (11.69-11.84) | 10.94 (10.77-11.11) | 10.80 (10.75-10.86) | 11.63 (11.58-11.69) |
| Chronic Renal Insufficiency | 5.60 (5.55-5.66) | 14.48 (14.30-14.65) | 5.41 (5.37-5.45) | 5.44 (5.40-5.48) |
| **Vital and Laboratory Measurements** |  |  |  |  |
| SBP mmHg (mean) | 157.02 (156.94-157.11) | N/A | 157.51 (157.43-157.60) | 157.35 (157.27-157.43) |
| BMI (mean) | 28.34 (28.32-28.36) | N/A | 28.37 (28.34-28.39) | 28.35 (28.33-28.37) |
| HbA1c-% (mean) | 6.71 (6.70-6.72) | N/A | 6.77 (6.76-6.78) | 6.74 (6.74-6.75) |
| Blood Glucose mg/dL (mean) | 142.48 (142.29-142.68) | N/A | 143.65 (143.45-143.85) | 143.42 (143.22-143.62) |
| Serum Creatinine mg/dL (mean) | 1.39 (1.38-1.41) | N/A | 1.44 (1.42-1.45) | 1.37 (1.36-1.38) |
| **Arrival Information** |  |  |  |  |
| Arrival Mode: EMS | 49.63 (49.51-49.75) | N/A | 48.88 (48.79-48.97) | 48.43 (48.34-48.52) |
| Ambulatory Status at Admission |  |  |  |  |
| Unable to ambulate | 32.84 (32.70-32.98) | N/A | 31.75 (31.65-31.86) | 31.79 (31.69-31.89) |
| With assistance from person | 27.37 (27.24-27.50) | N/A | 28.01 (27.91-28.11) | 28.28 (28.19-28.38) |
| Able to ambulate independently | 39.79 (39.64-39.94) | N/A | 40.24 (40.13-40.34) | 39.93 (39.82-40.03) |
| On-time Arrival (non-holiday weekday 7am-6pm) | 48.44 (48.32-48.55) | N/A | 49.00 (48.92-49.09) | 48.71 (48.63-48.80) |
| Initial NIHSS Score (0-42) (mean) | 6.70 (6.68-6.71) | N/A | 6.63 (6.61-6.65) | 6.68 (6.66-6.70) |
| **Medications Prior to Admission** |  |  |  |  |
| Antiplatelets | 49.64 (49.52-49.76) | N/A | 49.49 (49.40-49.57) | 49.15 (49.07-49.24) |
| Anticoagulants | 15.87 (15.77-15.98) | N/A | 15.49 (15.41-15.56) | 15.56 (15.49-15.64) |
| Antihypertensives | 69.26 (69.14-69.38) | N/A | 69.30 (69.22-69.39) | 69.14 (69.06-69.23) |
| Cholesterol-Reducers | 44.35 (44.23-44.46) | N/A | 43.98 (43.90-44.06) | 43.55 (43.46-43.63) |
| Diabetic Medications | 26.98 (26.86-27.09) | N/A | 27.61 (27.53-27.69) | 27.37 (27.29-27.45) |
| **Outcomes** |  |  |  |  |
| Length of Stay (mean days) | 5.18 (5.17-5.20) | 4.93 (4.89-4.97) | 5.03 (5.01-5.04) | 5.08 (5.07-5.09) |
| Discharge Disposition |  |  |  |  |
| Home | 47.26 (47.14-47.37) | 35.82 (35.56-36.07) | 47.79 (47.70-47.87) | 47.56 (47.48-47.64) |
| Home Hospice | 1.38 (1.35-1.41) | N/A | 1.39 (1.37-1.41) | 1.42 (1.40-1.44) |
| Hospice Facility | 3.16 (3.12-3.20) | N/A | 3.13 (3.10-3.16) | 3.14 (3.11-3.16) |
| Acute Care Facility | 2.03 (2.00-2.06) | 2.90 (2.80-3.01) | 2.40 (2.37-2.42) | 2.49 (2.47-2.52) |
| Other Health Care Facility | 40.93 (40.81-41.04) | 42.72 (42.47-42.98) | 40.25 (40.16-40.33) | 40.25 (40.17-40.33) |
| Left Against Medical Advice | 0.68 (0.66-0.70) | 0.77 (0.74-0.81) | 0.69 (0.68-0.71) | 0.68 (0.67-0.70) |
| Expired (in-hospital mortality) | 4.48 (4.43-4.53) | 4.50 (4.41-4.59) | 4.26 (4.22-4.29) | 4.37 (4.33-4.40) |
| Discharge Disposition - Other Facilities |  |  |  |  |
| Skilled Nursing Facility | 43.40 (43.23-43.58) | N/A | 44.57 (44.44-44.70) | 43.93 (43.80-44.06) |
| Inpatient Rehabilitation Facility | 52.60 (52.42-52.78) | N/A | 51.19 (51.05-51.32) | 51.61 (51.48-51.74) |
| Long Term Care Hospital | 2.14 (2.09-2.19) | N/A | 2.16 (2.12-2.20) | 2.28 (2.25-2.32) |
| Intermediate Care facility | 0.96 (0.92-0.99) | N/A | 1.12 (1.10-1.15) | 1.15 (1.13-1.18) |

**eTable 5:** Characteristics of ischemic stroke patients by year using raked post-stratification weighting by year to the U.S. Population.

|  | **2014** | **2013** | **2012** |
| --- | --- | --- | --- |
|  | N=475,655 | N=460,400 | N=452,240 |
| **Hospital Characteristics** |  |  |  |
| Census divisions |  |  |  |
| Division 1 New England | 20,850 (4.38) | 19,520 (4.24) | 19,590 (4.33) |
| Division 2 Mid-Atlantic | 65,235 (13.71) | 63,295 (13.75) | 61,515 (13.60) |
| Division 3 East North Central | 73,970 (15.55) | 71,000 (15.42) | 70,615 (15.61) |
| Division 4 West North Central | 31,370 (6.60) | 30,020 (6.52) | 29,565 (6.54) |
| Division 5 South Atlantic | 104,030 (21.87) | 100,810 (21.90) | 98,905 (21.87) |
| Division 6 East South Central | 38,790 (8.16) | 37,970 (8.25) | 37,805 (8.36) |
| Division 7 West South Central | 54,260 (11.41) | 52,770 (11.46) | 51,445 (11.38) |
| Division 8 Mountain | 25,145 (5.29) | 24,040 (5.22) | 23,610 (5.22) |
| Division 9 Pacific | 62,005 (13.04) | 60,975 (13.24) | 59,190 (13.09) |
| Hospital ownership |  |  |  |
| Government | 58,860 (12.37) | 53,905 (11.71) | 52,635 (11.64) |
| Private, Non-Profit | 353,460 (74.31) | 343,445 (74.60) | 337,605 (74.65) |
| Private, Investor-Owned | 63,335 (13.32) | 63,050 (13.69) | 62,000 (13.71) |
| Rural/teaching status |  |  |  |
| Rural | 42,315 (8.90) | 49,910 (10.84) | 50,695 (11.21) |
| Urban nonteaching | 126,080 (26.51) | 175,275 (38.07) | 175,615 (38.83) |
| Urban teaching | 307,260 (64.60) | 235,215 (51.09) | 225,930 (49.96) |
| Bed Size Categories |  |  |  |
| Small | 77,520 (16.30) | 53,365 (11.59) | 53,745 (11.88) |
| Medium | 140,385 (29.51) | 120,995 (26.28) | 118,025 (26.10) |
| Large | 257,750 (54.19) | 286,040 (62.13) | 280,470 (62.02) |
| Primary Stroke Center | 119,228 (25.07) | 110,173 (23.93) | 112,451 (24.87) |
| Comprehensive Stroke Center | 18,289 (3.85) | 14,703 (3.19) | 14,329 (3.17) |
| Number of Beds, Median (IQR) | 307 (198 - 480) | 302 (192 - 464) | 301 (191 - 456) |
| Annual Volume of IS Admissions, Median (IQR) | 212 (147 - 326) | 208 (143 - 317) | 205 (138 - 314) |
| **Patient Characteristics** |  |  |  |
| Age, Mean (SD) | 70.36 (19.44) | 70.46 (19.93) | 70.58 (20.79) |
| Age category |  |  |  |
| ≤60 | 120,606 (25.36) | 116,019 (25.20) | 114,310 (25.28) |
| 61-70 | 107,466 (22.59) | 103,032 (22.38) | 98,533 (21.79) |
| 71-80 | 112,956 (23.75) | 108,542 (23.58) | 105,737 (23.38) |
| >80 | 134,627 (28.30) | 132,807 (28.85) | 133,660 (29.56) |
| Female | 240,054 (50.47) | 233,759 (50.77) | 231,013 (51.08) |
| Race/Ethnicity |  |  |  |
| White | 317,535 (66.76) | 306,280 (66.52) | 301,575 (66.68) |
| Black | 74,965 (15.76) | 71,985 (15.64) | 70,500 (15.59) |
| Hispanic | 34,045 (7.16) | 33,175 (7.21) | 31,395 (6.94) |
| Asian & Pacific Islander | 12,295 (2.58) | 11,840 (2.57) | 10,800 (2.39) |
| Other | 36,815 (7.74) | 37,120 (8.06) | 37,970 (8.40) |
| Health Insurance Status |  |  |  |
| Private/VA/Champus/Other Insurance | 169,465 (42.82) | 161,245 (42.77) | 169,092 (43.48) |
| Medicaid | 45,405 (11.47) | 40,796 (10.82) | 41,001 (10.54) |
| Medicare | 154,761 (39.10) | 144,220 (38.26) | 147,455 (37.92) |
| Self-Pay/No Insurance | 23,394 (5.91) | 27,708 (7.35) | 28,807 (7.41) |
| **Medical History** |  |  |  |
| Atrial Fibrillation/Flutter | 85,704 (18.16) | 82,524 (18.24) | 79,489 (17.80) |
| Previous Stroke/TIA | 147,149 (31.18) | 142,264 (31.44) | 139,827 (31.30) |
| CAD/Prior Myocardial Infarction | 115,722 (24.52) | 112,068 (24.77) | 114,026 (25.53) |
| Diabetes Mellitus | 165,350 (35.04) | 156,521 (34.59) | 152,063 (34.04) |
| Peripheral Vascular Disease | 21,631 (4.58) | 21,408 (4.73) | 21,094 (4.72) |
| Hypertension | 361,007 (76.51) | 345,107 (76.27) | 343,231 (76.84) |
| Smoker | 88,139 (18.68) | 86,084 (19.03) | 84,771 (18.98) |
| Dyslipidemia | 216,141 (45.81) | 201,680 (44.57) | 197,498 (44.22) |
| Heart Failure | 44,101 (9.35) | 41,250 (9.12) | 39,677 (8.88) |
| Prosthetic Heart Valve | 5,318 (1.13) | 6,104 (1.35) | 5,336 (1.19) |
| Obesity/Overweight | 75,643 (16.03) | 56,443 (12.47) | 16,050 (3.59) |
| Chronic Renal Insufficiency | 35,530 (7.53) | 29,743 (6.57) | 8,909 (1.99) |
| **Vital and Laboratory Measurements** |  |  |  |
| SBP mmHg, Mean (SD) | 157.53 (40.31) | 157.54 (41.18) | 157.47 (43.67) |
| BMI, Median (IQR) | 27.4 (23.8 - 31.8) | 27.3 (23.8 - 31.6) | 27.2 (23.7 - 31.6) |
| HbA1c %, Mean (SD) | 6.76 (2.52) | 6.77 (2.54) | 6.79 (2.66) |
| Blood Glucose mg/dL, Mean (SD) | 143.94 (96.57) | 143.6 (98.77) | 143.43 (102.09) |
| Serum Creatinine mg/dL, Median (IQR) | 1 (0.8 - 1.3) | 1 (0.8 - 1.3) | 1 (0.8 - 1.3) |
| **Arrival Information** |  |  |  |
| Arrival Mode: EMS | 225,917 (48.62) | 199,241 (49.15) | 189,858 (48.91) |
| Ambulatory Status at Admission |  |  |  |
| Unable to ambulate | 83,737 (31.80) | 84,333 (31.61) | 90,635 (31.85) |
| With assistance from person | 72,286 (27.45) | 75,164 (28.17) | 80,746 (28.38) |
| Able to ambulate independently | 107,331 (40.76) | 107,304 (40.22) | 113,162 (39.77) |
| On-time Arrival (non-holiday weekday 7am-6pm) | 230,849 (48.53) | 225,467 (48.97) | 224,002 (49.53) |
| Initial NIHSS Score (0-42) |  |  |  |
| Median (IQR) | 4 (1 - 9) | 4 (1 - 9) | 4 (1 - 9) |
| Mean (SD) | 6.55 (10.09) | 6.65 (10.43) | 6.72 (10.87) |
| **Medications Prior to Admission** |  |  |  |
| Antiplatelets | 213,961 (50.58) | 200,373 (50.20) | 183,630 (47.54) |
| Anticoagulants | 46,843 (17.66) | 42,702 (17.05) | 42,066 (12.59) |
| Antihypertensives | 261,433 (68.69) | 256,113 (69.20) | 260,859 (70.03) |
| Cholesterol-Reducers | 212,149 (44.81) | 201,574 (44.07) | 193,525 (43.02) |
| Diabetic Medications | 102,086 (27.90) | 99,876 (27.71) | 100,296 (27.22) |
| **Outcomes** |  |  |  |
| Length of Stay (days), Median (IQR) | 4 (2 - 6) | 4 (2 - 6) | 4 (2 - 6) |
| Discharge Disposition |  |  |  |
| Home | 227,236 (47.77) | 219,735 (47.73) | 216,442 (47.86) |
| Home Hospice | 7,017 (1.48) | 6,301 (1.37) | 6,018 (1.33) |
| Hospice Facility | 14,846 (3.12) | 14,522 (3.15) | 14,043 (3.11) |
| Acute Care Facility | 11,106 (2.33) | 10,963 (2.38) | 11,234 (2.48) |
| Other Health Care Facility | 191,920 (40.35) | 185,324 (40.25) | 181,483 (40.13) |
| Left Against Medical Advice | 3,396 (0.71) | 3,324 (0.72) | 2,925 (0.65) |
| Expired | 19,763 (4.15) | 19,677 (4.27) | 19,668 (4.35) |

**eTable** **6:** Characteristics of ischemic stroke patients by year using the Bayesian (flat prior) post-stratification weighting model by year to the U.S. Population.

|  | **2014** | **2013** | **2012** |
| --- | --- | --- | --- |
|  | N=475,654 | N=460,400 | N=452,240 |
| **Hospital Characteristics** |  |  |  |
| Census divisions |  |  |  |
| Division 1 New England | 21,177 (4.45) | 19,689 (4.28) | 20,394 (4.51) |
| Division 2 Mid-Atlantic | 66,046 (13.89) | 63,473 (13.79) | 61,846 (13.68) |
| Division 3 East North Central | 74,183 (15.60) | 71,716 (15.58) | 71,177 (15.74) |
| Division 4 West North Central | 29,109 (6.12) | 27,876 (6.05) | 26,723 (5.91) |
| Division 5 South Atlantic | 107,114 (22.52) | 104,638 (22.73) | 102,588 (22.68) |
| Division 6 East South Central | 36,398 (7.65) | 35,315 (7.67) | 35,786 (7.91) |
| Division 7 West South Central | 55,617 (11.69) | 53,450 (11.61) | 51,038 (11.29) |
| Division 8 Mountain | 23,214 (4.88) | 22,131 (4.81) | 21,389 (4.73) |
| Division 9 Pacific | 62,796 (13.20) | 62,112 (13.49) | 61,299 (13.55) |
| Hospital ownership |  |  |  |
| Government | 51,771 (10.88) | 46,054 (10.00) | 44,760 (9.90) |
| Private, Non-Profit | 360,786 (75.85) | 353,294 (76.74) | 349,529 (77.29) |
| Private, Investor-Owned | 63,099 (13.27) | 61,051 (13.26) | 57,952 (12.81) |
| Rural/teaching status |  |  |  |
| Rural | 24,411 (5.13) | 30,455 (6.61) | 28,772 (6.36) |
| Urban nonteaching | 123,595 (25.98) | 175,806 (38.19) | 178,340 (39.43) |
| Urban teaching | 327,650 (68.88) | 254,139 (55.20) | 245,128 (54.20) |
| Bed Size Categories |  |  |  |
| Small | 67,778 (14.25) | 46,685 (10.14) | 45,383 (10.04) |
| Medium | 134,337 (28.24) | 112,957 (24.53) | 109,718 (24.26) |
| Large | 273,540 (57.51) | 300,758 (65.33) | 297,139 (65.70) |
| Primary Stroke Center | 109,300 (22.98) | 102,689 (22.30) | 106,675 (23.59) |
| Comprehensive Stroke Center | 20,574 (4.33) | 15,562 (3.38) | 16,530 (3.66) |
| Number of Beds, Median (IQR) | 361 (208 - 540) | 346 (204 - 532) | 342 (205 - 523) |
| Annual Volume of IS Admissions, Median (IQR) | 234 (144 - 374) | 227 (144 - 354) | 223 (141 - 345) |
| **Patient Characteristics** |  |  |  |
| Age, Mean (SD) | 70.1 (19.54) | 70.26 (20.04) | 70.53 (20.85) |
| Age category |  |  |  |
| ≤60 | 123,346 (25.93) | 118,295 (25.69) | 115,024 (25.43) |
| 61-70 | 107,769 (22.66) | 103,203 (22.42) | 98,093 (21.69) |
| 71-80 | 112,990 (23.75) | 107,811 (23.42) | 105,784 (23.39) |
| >80 | 131,551 (27.66) | 131,091 (28.47) | 133,340 (29.48) |
| Female | 238,800 (50.20) | 232,425 (50.48) | 230,055 (50.87) |
| Race/Ethnicity |  |  |  |
| White | 315,319 (66.29) | 305,173 (66.28) | 302,729 (66.94) |
| Black | 73,806 (15.52) | 71,117 (15.45) | 69,304 (15.32) |
| Hispanic | 34,558 (7.27) | 33,634 (7.31) | 31,626 (6.99) |
| Asian & Pacific Islander | 16,781 (3.53) | 14,515 (3.15) | 13,837 (3.06) |
| Other | 35,191 (7.40) | 35,961 (7.81) | 34,744 (7.68) |
| Health Insurance Status |  |  |  |
| Private/VA/Champus/Other Insurance | 174,433 (43.54) | 169,059 (43.80) | 172,509 (44.34) |
| Medicaid | 47,951 (11.97) | 41,947 (10.87) | 41,105 (10.57) |
| Medicare | 151,353 (37.78) | 143,049 (37.06) | 144,471 (37.13) |
| Self-Pay/No Insurance | 24,587 (6.14) | 29,423 (7.62) | 28,448 (7.31) |
| **Medical History** |  |  |  |
| Atrial Fibrillation/Flutter | 85,633 (18.17) | 83,203 (18.41) | 81,235 (18.23) |
| Previous Stroke/TIA | 144,855 (30.74) | 139,443 (30.86) | 139,124 (31.22) |
| CAD/Prior Myocardial Infarction | 115,342 (24.48) | 110,923 (24.55) | 113,013 (25.36) |
| Diabetes Mellitus | 164,585 (34.93) | 155,209 (34.35) | 149,570 (33.56) |
| Peripheral Vascular Disease | 21,639 (4.59) | 21,572 (4.77) | 21,345 (4.79) |
| Hypertension | 358,949 (76.18) | 342,035 (75.70) | 342,041 (76.75) |
| Smoker | 89,431 (18.98) | 85,941 (19.02) | 84,522 (18.97) |
| Dyslipidemia | 216,791 (46.01) | 200,145 (44.30) | 196,360 (44.06) |
| Heart Failure | 44,261 (9.39) | 42,149 (9.33) | 39,637 (8.89) |
| Prosthetic Heart Valve | 5,345 (1.13) | 6,557 (1.45) | 5,996 (1.35) |
| Obesity/Overweight | 82,105 (17.43) | 60,410 (13.37) | 16,704 (3.75) |
| Chronic Renal Insufficiency | 36,124 (7.67) | 29,515 (6.53) | 8,833 (1.98) |
| Medical History Panel Missing | 4,487 (0.94) | 8,571 (1.86) | 6,588 (1.46) |
| **Vital and Laboratory Measurements** |  |  |  |
| SBP mmHg, Mean (SD) | 157.41 (40.39) | 157.36 (41.26) | 157.28 (43.5) |
| BMI, Median (IQR) | 27.4 (23.8 - 31.7) | 27.3 (23.8 - 31.7) | 27.2 (23.7 - 31.6) |
| HbA1c %, Mean (SD) | 6.74 (2.6) | 6.75 (2.58) | 6.74 (2.62) |
| Blood Glucose mg/dL, Mean (SD) | 119 (101 - 158) | 119 (101 - 157) | 119 (101 - 157) |
| Serum Creatinine mg/dL, Median (IQR) | 144.53 (98.74) | 143.16 (98.92) | 142.57 (100.44) |
| SBP mmHg, Mean (SD) | 1 (0.8 - 1.3) | 1 (0.8 - 1.3) | 1 (0.8 - 1.3) |
| **Arrival Information** |  |  |  |
| Arrival Mode: EMS | 222,089 (47.86) | 198,780 (49.01) | 187,422 (48.51) |
| Ambulatory Status at Admission |  |  |  |
| Unable to ambulate | 85,106 (31.94) | 85,250 (31.37) | 91,133 (32.05) |
| With assistance from person | 73,732 (27.67) | 77,888 (28.66) | 81,035 (28.50) |
| Able to ambulate independently | 107,597 (40.38) | 108,656 (39.98) | 112,164 (39.45) |
| On-time Arrival (non-holiday weekday 7am-6pm) | 229,363 (48.22) | 224,158 (48.69) | 222,759 (49.26) |
| Initial NIHSS Score (0-42) |  |  |  |
| Median (IQR) | 4 (1 - 9) | 4 (1 - 9) | 4 (1 - 10) |
| Mean (SD) | 6.6 (10.09) | 6.69 (10.42) | 6.76 (10.8) |
| **Medications Prior to Admission** |  |  |  |
| Antiplatelets | 211,109 (49.89) | 200,048 (49.88) | 182,750 (47.59) |
| Anticoagulants | 46,448 (17.33) | 43,612 (17.18) | 42,831 (12.90) |
| Antihypertensives | 262,594 (68.38) | 260,125 (69.07) | 260,421 (70.02) |
| Cholesterol-Reducers | 209,774 (44.41) | 198,608 (43.51) | 191,706 (42.67) |
| Diabetic Medications | 102,438 (27.75) | 100,016 (27.42) | 98,668 (26.94) |
| **Outcomes** |  |  |  |
| Length of Stay (days), Median (IQR) | 4 (2 - 6) | 4 (2 - 6) | 4 (2 - 6) |
| Discharge Disposition |  |  |  |
| Home | 226,756 (47.67) | 218,145 (47.38) | 215,387 (47.63) |
| Home Hospice | 7,167 (1.51) | 6,302 (1.37) | 6,232 (1.38) |
| Hospice Facility | 14,692 (3.09) | 14,533 (3.16) | 14,307 (3.16) |
| Acute Care Facility | 11,155 (2.35) | 11,717 (2.54) | 11,723 (2.59) |
| Other Health Care Facility | 191,421 (40.24) | 185,738 (40.34) | 181,610 (40.16) |
| Left Against Medical Advice | 3,287 (0.69) | 3,262 (0.71) | 2,910 (0.64) |
| Expired | 20,764 (4.37) | 20,152 (4.38) | 19,733 (4.36) |

**eTable 7:** National Characteristics of ischemic stroke stratified by U.S. Division using Bayesian post-stratification weights for 2014.

| **Variable** | **Division 1** | **Division 2** | **Division 3** | **Division 4** | **Division 5** | **Division 6** | **Division 7** | **Division 8** | **Division 9** |
| --- | --- | --- | --- | --- | --- | --- | --- | --- | --- |
|  | N=21,177 | N=66,047 | N=74,183 | N=29,109 | N=107,114 | N=36,398 | N=55,617 | N=23,214 | N=62,796 |
| **Patient Demographics** |  |  |  |  |  |  |  |  |  |
| Age | 74 (63 - 84) | 73 (62 - 83) | 72 (61 - 82) | 70 (59 - 81) | 70 (59 - 81) | 70 (59 - 80) | 69 (58 - 80) | 71 (60 - 81) | 72 (61 - 83) |
| Female | 10,787 (50.94) | 33,895 (51.32) | 37,619 (50.71) | 14,092 (48.41) | 54,043 (50.45) | 17,884 (49.13) | 27,981 (50.31) | 11,620 (50.06) | 30,880 (49.17) |
| Race/Ethnicity |  |  |  |  |  |  |  |  |  |
| ' White | 15,456 (72.98) | 45,809 (69.36) | 55,143 (74.33) | 17,020 (58.47) | 66,810 (62.37) | 28,151 (77.34) | 32,855 (59.07) | 14,524 (62.56) | 39,550 (62.98) |
| ' Black | 1,908 (9.01) | 10,419 (15.78) | 10,190 (13.74) | 2,749 (9.44) | 25,888 (24.17) | 5,002 (13.74) | 10,423 (18.74) | 1,856 (7.99) | 5,370 (8.55) |
| ' Hispanic | 1,424 (6.72) | 4,036 (6.11) | 2,580 (3.48) | 998 (3.43) | 5,931 (5.54) | 1,260 (3.46) | 6,819 (12.26) | 2,687 (11.58) | 8,822 (14.05) |
| ' Asian & PI | 1,051 (4.96) | 1,848 (2.80) | 1,777 (2.40) | 724 (2.49) | 2,473 (2.31) | 658 (1.81) | 1,730 (3.11) | 1,201 (5.17) | 5,319 (8.47) |
| ' Other | 1,338 (6.32) | 3,934 (5.96) | 4,493 (6.06) | 7,617 (26.17) | 6,012 (5.61) | 1,326 (3.64) | 3,789 (6.81) | 2,946 (12.69) | 3,735 (5.95) |
| Insurance [mod] |  |  |  |  |  |  |  |  |  |
| ' Private/VA/Champus/Other Insurance | 3,944 (23.45) | 13,353 (23.69) | 15,149 (22.69) | 6,088 (23.87) | 20,827 (22.45) | 5,549 (19.91) | 10,867 (24.08) | 5,148 (27.65) | 12,208 (25.17) |
| ' Medicaid | 1,415 (8.42) | 4,391 (7.79) | 4,603 (6.89) | 1,289 (5.05) | 6,556 (7.07) | 1,376 (4.94) | 2,861 (6.34) | 1,792 (9.63) | 4,583 (9.45) |
| ' Medicare | 11,097 (65.98) | 36,408 (64.60) | 44,206 (66.21) | 16,560 (64.93) | 57,750 (62.26) | 18,447 (66.18) | 26,630 (59.01) | 10,574 (56.80) | 29,767 (61.37) |
| ' Self Pay/No Insurance | 362 (2.15) | 2,207 (3.92) | 2,807 (4.20) | 1,568 (6.15) | 7,625 (8.22) | 2,501 (8.97) | 4,767 (10.56) | 1,102 (5.92) | 1,948 (4.02) |
| Initial NIHSS Score (0-42) | 3 (1 - 9) | 3 (1 - 8) | 3 (1 - 9) | 4 (1 - 9) | 4 (1 - 9) | 4 (1 - 9) | 4 (1 - 10) | 4 (1 - 10) | 4 (1 - 10) |
| AF history/PAF | 5,833 (27.59) | 16,054 (24.32) | 17,204 (23.23) | 6,957 (24.24) | 23,964 (22.39) | 7,726 (21.23) | 10,800 (19.43) | 5,244 (22.85) | 15,898 (25.33) |
| **Medical History** |  |  |  |  |  |  |  |  |  |
| Atrial Fibrillation/Flutter | 4,600 (21.83) | 12,587 (19.13) | 13,675 (18.56) | 5,064 (17.77) | 18,824 (17.71) | 5,823 (16.03) | 8,316 (15.17) | 3,916 (17.82) | 12,826 (20.47) |
| Previous Stroke/TIA | 5,965 (28.31) | 20,130 (30.60) | 23,097 (31.34) | 7,873 (27.63) | 33,007 (31.05) | 11,607 (31.96) | 17,170 (31.31) | 6,649 (30.25) | 19,358 (30.89) |
| CAD/Prior MI | 4,974 (23.60) | 16,534 (25.13) | 19,063 (25.87) | 6,891 (24.18) | 26,180 (24.63) | 10,037 (27.63) | 13,157 (23.99) | 4,832 (21.99) | 13,673 (21.82) |
| Diabetes Mellitus | 6,682 (31.71) | 22,781 (34.63) | 25,281 (34.30) | 9,709 (34.07) | 38,184 (35.92) | 12,931 (35.60) | 20,659 (37.68) | 7,013 (31.91) | 21,345 (34.06) |
| Peripheral Vascular Disease (PVD) | 1,054 (5.00) | 2,932 (4.46) | 4,271 (5.80) | 1,276 (4.48) | 4,771 (4.49) | 1,814 (5.00) | 1,756 (3.20) | 813 (3.70) | 2,951 (4.71) |
| Hypertension | 15,971 (75.79) | 50,409 (76.63) | 56,393 (76.52) | 20,501 (71.94) | 82,348 (77.46) | 28,083 (77.32) | 42,425 (77.37) | 15,616 (71.06) | 47,202 (75.32) |
| Smoker | 3,255 (15.45) | 10,904 (16.57) | 15,717 (21.33) | 5,938 (20.84) | 21,922 (20.62) | 8,094 (22.29) | 11,190 (20.41) | 3,557 (16.19) | 8,854 (14.13) |
| Dyslipidemia | 11,207 (53.18) | 31,020 (47.15) | 36,846 (50.00) | 13,316 (46.72) | 49,554 (46.61) | 14,985 (41.26) | 22,259 (40.59) | 9,458 (43.04) | 28,147 (44.91) |
| Heart Failure | 2,122 (10.07) | 5,655 (8.60) | 7,989 (10.84) | 2,371 (8.32) | 9,459 (8.90) | 3,511 (9.67) | 5,388 (9.83) | 1,368 (6.22) | 6,400 (10.21) |
| Prosthetic Heart Valve | 253 (1.20) | 981 (1.49) | 909 (1.23) | 308 (1.08) | 1,168 (1.10) | 309 (0.85) | 479 (0.87) | 264 (1.20) | 674 (1.08) |
| Obesity/Overweight | 2,980 (14.14) | 10,633 (16.16) | 15,246 (20.69) | 4,629 (16.24) | 20,128 (18.93) | 5,683 (15.65) | 6,798 (12.40) | 4,975 (22.64) | 11,034 (17.61) |
| Chronic Renal Insufficiency | 1,750 (8.30) | 4,542 (6.90) | 5,667 (7.69) | 2,139 (7.51) | 8,208 (7.72) | 2,250 (6.20) | 3,635 (6.63) | 1,531 (6.97) | 6,402 (10.21) |
| **Medications Prior to Admission** |  |  |  |  |  |  |  |  |  |
| Antiplatelets | 9,628 (53.33) | 31,129 (50.87) | 36,656 (54.32) | 13,736 (52.54) | 46,026 (48.65) | 16,978 (50.88) | 22,137 (45.55) | 9,111 (45.96) | 25,707 (47.71) |
| Anticoagulants | 2,212 (20.34) | 7,747 (19.89) | 7,810 (19.47) | 2,940 (17.34) | 9,701 (16.40) | 3,662 (17.43) | 4,175 (13.16) | 2,119 (16.07) | 6,080 (16.88) |
| Antihypertensives | 12,045 (71.32) | 41,818 (70.38) | 44,873 (69.50) | 13,607 (65.98) | 61,477 (69.17) | 18,814 (70.50) | 28,026 (65.96) | 12,754 (64.00) | 29,179 (65.47) |
| Cholesterol-Reducers | 10,493 (49.99) | 30,403 (46.11) | 35,051 (47.42) | 12,351 (42.96) | 45,983 (43.72) | 15,779 (43.38) | 22,659 (40.76) | 9,510 (41.22) | 27,543 (44.05) |
| Diabetic Medications | 4,243 (26.58) | 16,054 (27.86) | 16,789 (27.67) | 5,577 (28.61) | 24,576 (28.50) | 7,400 (27.94) | 11,291 (28.80) | 5,133 (25.81) | 11,374 (26.07) |
| **Outcomes** |  |  |  |  |  |  |  |  |  |
| Discharge Disposition |  |  |  |  |  |  |  |  |  |
| ' Home | 9,036 (42.67) | 29,098 (44.06) | 34,164 (46.05) | 13,630 (46.82) | 53,776 (50.20) | 18,388 (50.52) | 26,643 (47.90) | 11,050 (47.60) | 30,970 (49.32) |
| ' Hospice = Home | 223 (1.05) | 730 (1.10) | 992 (1.34) | 449 (1.54) | 1,567 (1.46) | 478 (1.31) | 790 (1.42) | 405 (1.75) | 1,534 (2.44) |
| ' Hospice = Health Care Facility | 535 (2.53) | 1,971 (2.98) | 2,824 (3.81) | 688 (2.36) | 4,002 (3.74) | 840 (2.31) | 1,802 (3.24) | 975 (4.20) | 1,054 (1.68) |
| ' Acute Care Facility | 615 (2.90) | 1,409 (2.13) | 1,464 (1.97) | 640 (2.20) | 2,725 (2.54) | 362 (1.00) | 1,331 (2.39) | 543 (2.34) | 2,065 (3.29) |
| ' Other Health Care Facility | 9,529 (45.00) | 29,361 (44.45) | 31,364 (42.28) | 12,174 (41.82) | 40,105 (37.44) | 13,984 (38.42) | 22,183 (39.89) | 9,128 (39.32) | 23,594 (37.57) |
| ' Expired | 1,079 (5.10) | 2,948 (4.46) | 3,034 (4.09) | 1,320 (4.54) | 3,983 (3.72) | 2,119 (5.82) | 2,443 (4.39) | 874 (3.77) | 2,964 (4.72) |
| ' Left Against Medical Advice/AMA | 158 (0.75) | 482 (0.73) | 332 (0.45) | 162 (0.56) | 856 (0.80) | 196 (0.54) | 412 (0.74) | 194 (0.84) | 494 (0.79) |
| Discharge Disposition - Other Facilities |  |  |  |  |  |  |  |  |  |
| ' Skilled Nursing Facility (SNF) | 3,977 (42.49) | 11,749 (40.03) | 13,541 (43.27) | 4,581 (37.66) | 19,035 (48.71) | 6,180 (44.21) | 7,167 (32.37) | 3,512 (39.30) | 13,342 (56.67) |
| ' Inpatient Rehabilitation Facility (IRF) | 5,000 (53.43) | 16,848 (57.40) | 16,120 (51.51) | 6,903 (56.75) | 18,816 (48.15) | 7,213 (51.60) | 13,120 (59.26) | 5,048 (56.50) | 9,493 (40.32) |
| ' Long Term Care Hospital (LTCH) | 277 (2.96) | 313 (1.06) | 699 (2.23) | 194 (1.60) | 646 (1.65) | 342 (2.45) | 890 (4.02) | 232 (2.60) | 435 (1.85) |
| ' Intermediate Care facility (ICF) | 58 (0.62) | 234 (0.80) | 576 (1.84) | 284 (2.34) | 255 (0.65) | 152 (1.09) | 582 (2.63) | 77 (0.86) | 140 (0.59) |
| **GWTG Hospital Characteristics** |  |  |  |  |  |  |  |  |  |
| Primary Stroke Center | 8,008 (37.82) | 34,097 (51.63) | 60,760 (81.90) | 21,280 (73.10) | 77,462 (72.32) | 25,759 (70.77) | 37,204 (66.89) | 19,252 (82.93) | 45,951 (73.17) |
| Comprehensive Stroke Center | 1,845 (8.71) | 11,381 (17.23) | 15,844 (21.36) | 3,537 (12.15) | 15,363 (14.34) | 7,816 (21.47) | 5,401 (9.71) | 1,079 (4.65) | 4,786 (7.62) |
| Rural location | 1,173 (5.54) | 1,273 (1.93) | 3,698 (4.98) | 2,467 (8.47) | 6,603 (6.17) | 2,426 (6.67) | 3,944 (7.09) | 735 (3.17) | 2,093 (3.33) |
| Academic Hospital | 15,930 (75.22) | 54,312 (82.23) | 54,730 (73.78) | 21,800 (74.89) | 68,553 (64.00) | 26,661 (73.25) | 37,779 (67.94) | 15,826 (68.17) | 38,511 (61.33) |

**eFigure 1:** Distribution of raking post-stratification weights stratified by year.

**eFigure 2:** Distribution of Bayesian post-stratification weights stratified by year.
